# Impact of 50 g OGTT-Induced Acute Hyperglycemia on Uteroplacental and Fetoplacental Circulations in Gestational Diabetes Screening: A Prospective Single-Center Study

**DOI:** 10.3390/jcm14238362

**Published:** 2025-11-25

**Authors:** Hilal Gülsüm Turan Özsoy, Gültekin Adanaş Aydın, Serhat Ünal, Behiye Oral

**Affiliations:** Bursa City Hospital, 16110 Bursa, Turkey; h.g.turan@hotmail.com (H.G.T.Ö.); serhatunaldr@yahoo.com (S.Ü.); behiyeoral@gmail.com (B.O.)

**Keywords:** gestational diabetes, oral glucose tolerance test, Doppler ultrasonography, fetoplacental circulation, uteroplacental circulation

## Abstract

**Background:** Gestational diabetes screening often employs the 50 g oral glucose tolerance test (OGTT), which induces acute hyperglycemia. This study aimed to investigate the effects of this acute hyperglycemia on uteroplacental and fetoplacental circulations in women between the 24th and 28th weeks of pregnancy using Doppler ultrasonography (USG). **Methods:** This prospective, single-center study included 209 pregnant women with singleton pregnancies followed at Bursa City Hospital between January and April 2021. Doppler USG measurements were taken before and 60 min after administering the 50 g OGTT. Venous blood samples were collected to measure blood glucose levels pre-test and one hour post-test. Doppler indices, including the pulsatility index (PI), resistive index (RI), and systolic/diastolic ratio (S/D) for the uterine arteries, umbilical artery (UA), and middle cerebral artery (MCA), were recorded and converted to Z scores based on gestational age. Fetal biometric parameters, including abdominal circumference (AC), femur length (FL), and biparietal diameter (BPD), were also measured. **Results:** The study found a significant decrease in the mean MCA-PI, MCA RI, MCA S/D, and cerebroplacental ratio (CPR) values, as well as their respective Z scores, in the post-test measurements compared to pre-test values (*p* < 0.001 for all). No significant differences were observed in the S/D, RI, PI values, or Z scores of the uterine arteries and UA between pre-test and post-test measurements. **Conclusions:** Acute hyperglycemia induced by the 50 g OGTT significantly affects fetal cerebral circulation, evidenced by decreased MCA-PI, MCA RI, MCA S/D, and CPR values, but does not significantly impact uteroplacental circulation. Further large-scale studies are necessary to explore the effects of varying maternal glucose levels and the chronic impacts of non-physiological glucose levels on placental circulation.

## 1. Introduction

In obstetrics, Doppler ultrasonography (USG) is widely used as a reliable examination method to assess fetal well-being, which depends on several factors, including maternal nutritional intake, maternal body composition, uteroplacental and fetoplacental blood flow, placental transfer and nutrient metabolism.

The relationship between placental insufficiency and possible outcomes such as perinatal death, intrauterine growth retardation (IUGR), and preeclampsia (PE) reveals the importance of Doppler USG in obstetrics.

Given the increase in the distribution of cardiac output towards the brain (cerebral redistribution) during fetal hypoxia, Doppler USG can assess the response of the relevant organ to fetal hypoxia. Increased cerebral blood flow leads to a decrease in the middle cerebral artery (MCA) resistive index (RI) and an increase in the umbilical artery (UA) RI, a phenomenon known as the brain-sparing effect [1,2,3]. A decrease in the cerebroplacental ratio (CPR), calculated by dividing the MCA pulsatility index (PI) by the UA PI, is an early indicator of fetal hypoxia and is associated with adverse pregnancy outcomes and the need for therapeutic delivery in term fetuses [4,5].

The UA PI assesses placental functionality, with higher resistance being associated with placental insufficiency and intrauterine growth retardation [6]. Both uteroplacental and fetoplacental circulations can be evaluated via Doppler USG scans of the uterine and umbilical arteries. CPR effectively reflects placental health and fetal response.

Transport of glucose, the primary energy source for the fetus, across the placenta via facilitated diffusion depends on the mother’s blood sugar levels and placental blood flow [7]. Some studies assessing Doppler indices before and after oral glucose tolerance test (OGTT) reported a decrease in MCA-PI, while others reported no significant change [7,8,9,10]. Similarly, some studies reported no significant change in UA-PI after OGTT, while others reported an increase or decrease [7,8,9,10].

In light of this information, this study was conducted to investigate the impact of acute hyperglycemia induced by 50-g OGTT, which is commonly used for gestational diabetes screening, on uteroplacental and fetoplacental circulations in women between the 24th and 28th weeks of gestation, using Doppler USG.

## 2. Materials and Methods

### 2.1. Study Design

This study was designed as a prospective single-center study. The study protocol was approved by the Bursa City Hospital Ethics Committee (Decision No: 2021-11/3). The study was conducted in accordance with the ethical principles outlined in the Declaration of Helsinki. Written informed consent was obtained from the participants included in the study.

### 2.2. Population and Sample

The study population consisted of women between the 24th and 28th weeks of pregnancy who had singleton pregnancies followed at Bursa City Hospital Obstetrics and Gynecology and Radiology Clinics between 18 June 2021–18 August 2021. Demographic and clinical characteristics of the patients, including maternal age, parity, gravidity, abortion history, gestational diabetes history, familial diabetes mellitus (DM) history, presence of systemic diseases, smoking history, gestational age, and last menstrual period, were recorded. The gestational age of participants whose last menstrual period was unknown was determined based on the crown-rump length (CRL) obtained from USG measurements carried out in the first trimester. Patients with hypertension, DM, renal and endocrinological diseases, those using drugs that could affect fetoplacental and uteroplacental Doppler indices, those with fetal anomalies, and those with multiple pregnancies were excluded from the study. Patients with normal fasting blood glucose levels and without impaired glucose tolerance in the first trimester were included in the study. In the end, the study sample consisted of 209 women.

### 2.3. OGTT and Doppler USG Procedures

All participants underwent 50-g OGTT as part of gestational diabetes screening. Venous blood samples were taken from the patients, and their blood sugar levels were measured twice before the test (first measurement) and one hour after the test (second measurement).

Doppler USG scans were performed while the patients were on an empty stomach, rested, and remained seated at 08:30 a.m. before OGTT and 60 min after the OGTT. All Doppler USG scans were conducted by a radiologist with 13 years of experience in Doppler USG, using a Voluson E8 (GE Healthcare, Austria GmbH &. Co OG, Tiefenbach, Austria) device equipped with an electronic matrix convex probe (2–9 MHz).

Intra-observer reliability results showed high agreement between right and left uterine artery measurements (*p* < 0.0001, Table 1).

### 2.4. Analysis of Doppler Waves

Within the scope of the analysis of Doppler waves, the uterine artery, UA, and MCA indices shown below, calculated independently of the angle of the sound wave or flow angle, were measured.

The UA parameters were measured at the free loop of the umbilical cord in the absence of respiration or fetal movement. During each measurement, 10 to 15 waveforms were evaluated in three consecutive waveforms. The MCA parameters were measured at the proximal MCA of the cerebral hemisphere closer to the probe, at the starting point close to the circle of Willis. The CPR was calculated. The MCA and UA Doppler indices were measured at a fetal heart rate (FHR) of 130–150 bpm. Uterine artery Doppler assessment, each artery was identified using color flow mapping at the crossover with the external iliac artery. All Doppler indices were measured per the International Society of Ultrasound in Obstetrics and Gynecology (ISUOG) guidelines [11]. Measurements were made with an insonation angle of less than 30°, avoiding any maternal or fetal movement. At least three consecutive waveforms or a 10-s automatic trace were used for umbilical vein (UV) flow. Measurements were not performed after uterine contractions or fetal deceleration to avoid potential pathological waveforms [12].

Since Doppler indices can vary with gestational age, the values were converted to Z scores. The Z scores, calculated using the formula of observed value—expected value for gestational age/standard deviation (SD) for gestational age, represent the deviation from the mean in units of SD [13,14]. The Z scores for MCA-PI, UA-PI, CPR, and uterine artery-PI were calculated electronically (https://fetalmedicine.org/research/doppler, accessed on 14 May 2025). Participants’ fetal biometric parameters, such as abdominal circumference (AC), femur length (FL), and biparietal diameter, were also measured.

### 2.5. Statistical Analysis

Statistical analyses were conducted using SPSS software, version 22.0 (IBM Corp., Armonk, NY, USA). Descriptive statistics were presented as mean ± standard deviation (SD), interquartile range (IQR), as well as absolute numbers and percentages where appropriate. The distribution of continuous variables was assessed by applying the Kolmogorov–Smirnov test in both the first and second measurements in order to determine whether the data conformed to a normal distribution. For comparisons between the left and right indices obtained during the initial and subsequent measurements, the Wilcoxon signed-rank test was employed, given its suitability for paired non-parametric data. In addition, the associations between measurement outcomes and quantitative variables were examined using Spearman’s rank correlation analysis, which is particularly appropriate for evaluating monotonic relationships without assuming normality. A *p*-value of less than 0.05 was accepted as the threshold for statistical significance throughout the analysis.

## 3. Results

A total of 209 pregnant women with a mean age of 28.7 ± 5.38 (min. 19, max. 41) years were included in the study (Table 2). Of the 209 participants, 23 (11%) had a systemic disease history (3 patients with asthma, 1 patient with Behçet’s disease, and 7 patients with hypothyroidism), 28 (13.4%) had smoking history, 15 (7.2%) had regular drug use, 63 (30.1%) had familial DM history, 5 (2.4%) had a history of DM in a previous pregnancy, 7 (3.4%) had a history of preeclampsia, 11 (5.3%) had a history of IUGR in an earlier pregnancy and 10 (4.8%) had a history of preterm delivery (Table 3).

There was no significant difference in the right and left uterine artery S/D, RI, PI values, and PI-Z scores between the first and second measurements (right uterine artery; *p* = 0.107, *p* = 0.437, *p* = 0.189, and *p* = 0.412, respectively, and left uterine artery; *p* = 0.375, *p* = 0.566, *p* = 0.387, and *p* = 0.464, respectively). Given the lack of significant difference in the right and left uterine artery S/D, RI, PI values, and PI-Z scores between the first and second measurements, the mean values of the uterine artery indices were compared between the first and second measurements. However, no significant difference was found in mean S/D, RI, PI values, or PI-Z scores of the uterine artery between the first and second measurements (*p* = 0.129, *p* = 0.411, *p* = 0.200, and *p* = 0.284, respectively).

There was also no significant difference in UA’s S/D, RI, PI values, or PI-Z scores between the first and second measurements (*p* = 0.898, *p* = 0.900, *p* = 0.901, and *p* = 0.947, respectively).

The mean MCA/PI, MCA RI, MCA S/D, CPR values, MCA/PI, and CPR Z scores obtained from the second measurement were significantly lower than those obtained from the first measurement (*p* < 0.001, *p* < 0.001, *p* < 0.001, *p* = 0.004, *p* < 0.001, and *p* = 0.003, respectively) (Table 4).

Patients with a 50 g glucose challenge test result of ≥140 mg/dL underwent the 100 g OGTT. Doppler ultrasonography parameters of patients with a 50 g glucose test ≥140 mg/dL were compared. The comparison of patients with positive (n = 62) and negative (n = 147) screening results is presented in Table 5. Examination of Table 5 shows that in patients with a positive screening test, left uterine artery PI, RI, and S/D values decreased in the second measurements, whereas in patients with a negative screening test, these values increased. No significant differences were observed between measurements for other Doppler parameters outside of the left uterine artery.

Similarly, Table 6 presents the comparison of patients with positive (n = 7) and negative (n = 174) results of the 100 g OGTT. In patients with a positive OGTT result, left uterine artery PI, RI, and S/D values decreased in the second measurements, whereas in patients with a negative result, these values increased. No significant differences were observed for other Doppler parameters outside of the left uterine artery.

## 4. Discussion

This study investigated the impact of acute hyperglycemia induced by 50-g OGTT, used for gestational diabetes screening, on fetoplacental and uteroplacental circulation in women between the 24th and 28th weeks of gestation using Doppler USG. Consequently, it was found that mean MCA/PI, MCA RI, MCA S/D, CPR values, MCA/PI, and CPR Z scores values obtained from the second measurement (after OGTT) were significantly lower than those obtained from the first measurement (before OGTT).

In a study in which UA and MCA velocity waveforms were measured using Doppler USG before and two hours after the 75-g OGTT in 105 low-risk pregnant women between the 30th and 32nd weeks of gestation, Haugen et al. found that the MCA-PI decreased significantly following the OGTT, independently of the fetal heart rate (FHR). They also reported that the UA-PI value decreased significantly after the test; however, they noted that these changes were related to FHR [7]. They concluded that the maternal glucose challenge test can reduce fetal cerebral blood flow impedance, regardless of fetal size.

In another study, Opheim and colleagues [15] investigated the effect of maternal nutrition on Doppler blood flow velocity variables in 89 women between the 30th and 36th weeks of gestation with normal singleton pregnancies. They performed the first measurements, including UA-PI, MCA-PI, PSV, and FHR, at 08:30 a.m. while the patients were on an empty stomach. The participants were then given a standard breakfast with approximately 400 kcal of energy content. The second measurements were performed 105 min after breakfast. Consequently, they found that the fetal MCA-PI value had decreased significantly, independent of the FHR. They did not report any significant postprandial change in the UA-PI value, except for male fetuses at 30 weeks. On the other hand, they reported a significant postprandial decrease in CPR value and a significant increase in the MCA-PSV value [7].

In a study investigating the impact of glucose on Doppler flow velocity in the MCA and UA of 24 healthy pregnant women between the 36th and 40th weeks of gestation, Gillis et al. [8] found a significant decrease in the RI value of both arteries after glucose challenge test. In a study investigating fetal MCA and UA blood flow before and after the 50-g OGTT in pregnant women, Pardo et al. [16] found a significant decrease in MCA-RI value and a significant increase in UA-RI value.

Zahid et al. conducted a study involving 25 participants at 36 weeks of gestation, dividing them into two groups. Doppler ultrasonography measurements were performed at approximately 8:30 a.m. while all participants were fasting. One group was provided a 400 kcal breakfast, whereas the other group remained fasting. After 1–3 days, the groups were switched, and measurements were repeated. The MCA PI and UA PI values, both adjusted and unadjusted for fetal heart rate (FHR), did not differ significantly between the two meal states. This study is noteworthy, as it included a control group—unlike many previous reports—and the authors attributed the absence of significant differences in MCA PI values between groups to diurnal variations [17].

In our study, because Doppler parameters are known to be affected by FHR, fetal respiration, and movements, all measurements were performed during periods when the fetus was more stable. However, adjusted comparisons considering FHR were not performed. Instead, the measurements were obtained when the FHR was within the 130–150 bpm range, aiming to minimize the potential influence of FHR on Doppler indices. As noted in this paragraph, since our study did not include a control group, we were unable to determine whether the observed changes in MCA PI were due to glycemic fluctuations or diurnal variation.

Degani et al. [10] reported increased PI values of the UA and fetal intracranial internal carotid artery one hour after the 100-g OGTT, indicating elevated cerebrovascular resistance due to increased glucose levels. A study conducted with 30 healthy pregnant women between the 24th and 28th weeks of gestation found no significant difference in the S/D and RI values of the UA and uterine artery before and one, two, and three hours after the 100-g OGTT [9]. Similarly, several other studies found no significant change in the UA-PI value after OGTT [8,9,18]. Along these lines, some studies have shown no change in MCA-PI and fetal AC values or AC Z scores due to OGTT [7,15].

Although several studies [7,8,15,16] reported a decrease in the MCA-PI values after the maternal glucose challenge test featuring non-physiological doses, overall, the results of the studies on the impact of the increase in non-physiological maternal glucose levels on fetoplacental and uteroplacental circulation can be considered controversial.

In line with the literature, we found the mean MCA/PI, MCA RI, MCA S/D, CPR values, MCA/PI, and CPR Z scores obtained from the second measurement were significantly lower than those obtained from the first measurement. In parallel with other studies in the literature, the significant decrease we detected in CPR value and CPR Z score was an expected finding, given the decrease in the MCA PI, MCA RI, MCA S/D values and MCA PI-Z score.

Plot MCA-PI vs. gestational age graph is parabolic, featuring a gradual decrease in MCA-PI value in the third trimester [19]. This decrease is associated with a nearly threefold increase in brain weight [20], which leads to higher energy demands and enhanced vascularization in the fetal brain [15,21]. The postprandial decrease in MCA-PI supports brain development by increasing blood and nutrient flow to the brain [16]. Vasodilation is thought to contribute to reducing cerebral vascular resistance, potentially through neural mechanisms and circulating vasoactive agents. One of these mechanisms is the endothelium-dependent L-arginine/nitric oxide pathway [22,23]. Elevated glucose levels within the normal range in human umbilical vein endothelial cell cultures reduce vessel wall tension by stimulating insulin-induced nitric oxide production [24]. High glucose levels also activate nicotinamide adenine dinucleotide phosphate oxidase (NOX), generating significant amounts of reactive oxygen species [24]. Insulin protects endothelial functions against high glucose concentrations by increasing nitric oxide synthesis via human cationic amino acid transporter-1 (hCAT-1). It has been reported that at high D-glucose concentrations, tetrahydrobiopterin levels in human umbilical vein endothelial cells (HUVECs) are not restored by insulin, leading to increased vasoconstriction and decreased vasodilation with higher oxidative stress [24]. Mancusi et al. [25] found no significant difference in the enzyme’s expression, intracellular distribution, or basal activity at either incubation concentration.

Our study included a more significant number of patients than all other relevant studies in the literature. Intra-observer reliability analysis revealed high agreement between the first and second measurements, indicating that our measurements were reliable. Additionally, considering that Doppler indices change according to gestational age, calculating the Z scores of these indices adds more value to our study. To our knowledge, our study is one of the few studies comparing Z scores in this patient population. Our study did not include a control group; we were unable to determine whether the observed changes in MCA PI were due to glycemic fluctuations or diurnal variation.

In conclusion, understanding the impact of maternal glucose levels and insulin on fetoplacental circulation is crucial, given the complexity of the mechanisms involved. Previous studies have often used relatively small samples of pregnant women at varying gestational ages, making it difficult to draw definitive conclusions. Our findings indicate that acute hyperglycemia induced by OGTT significantly affects fetal cerebral circulation, as evidenced by lower MCA/PI, CPR values, and MCA/PI and CPR Z scores, but not uteroplacental circulation. Further large-scale studies are needed to explore different maternal glucose levels and the chronic effects of non-physiological maternal glucose values.

## Figures and Tables

**Table 1:** Intra-Observer Reliability Results for Uterine Artery Measurements.

Measurement	Intraclass Correlation (ICC)	Lower Bound	Upper Bound	*p*-Values
R UT S/D	0.970	0.944	0.985	<0.001
R UT PI	0.967	0.937	0.983	<0.001
R UT RI	0.924	0.858	0.960	<0.001
L UT S/D	0.943	0.894	0.970	<0.001
L UT PI	0.944	0.895	0.971	<0.001
L UT RI	0.961	0.927	0.980	<0.001

Abbreviations: R UT: Right Uterine Artery, L UT: Left Uterine Artery, S/D: Systolic/Diastolic Ratio, PI: Pulsatility Index, RI: Resistive Index, ICC: Intraclass Correlation.

**Table 2:** Descriptive Statistics for Study Variables.

Variable	n	Mean ± SD	Median [Min–Max]	25th–75th Percentiles
Age (years)	207	28.71 ± 5.39	28.0 [19–41]	[25.0–32.0]
Height (cm)	209	162.09 ± 5.59	162.0 [150.0–178.0]	[158.5–166.0]
Weight (kg)	209	75.99 ± 13.06	74.0 [48.0–116.0]	[67.0–84.5]
BMI (kg/m^2^)	209	28.96 ± 4.99	28.20 [19.47–43.66]	[25.23–32.30]
Pre-pregnancy weight (kg)	209	68.01 ± 13.28	65.0 [40.0–113.0]	[58.5–75.0]
Gestational week (LMP)	202	25.43 ± 1.84	25.14 [18.71–30.71]	[24.14–26.61]
Gestational week (US)	209	25.77 ± 1.98	25.43 [19.14–32.14]	[24.50–27.07]
Birth weight, 1st delivery (g)	128	3205.63 ± 532.77	3200.0 [700–4550]	[2910.0–3590.0]
Birth weight, 2nd delivery (g)	55	3319.27 ± 391.78	3300.0 [2500–4300]	[3050.0–3600.0]
Birth weight, 3rd delivery (g)	15	3490.0 ± 562.58	3300.0 [2500–4650]	[3100.0–4000.0]
USG/BPD (cm)	209	25.81 ± 2.22	25.40 [19.5–32.0]	[24.35–27.30]
AC (cm)	209	25.68 ± 1.99	25.50 [19.3–32.6]	[24.30–27.05]
FL (cm)	209	25.43 ± 2.14	25.30 [19.0–31.4]	[24.20–26.55]
EFW (g)	209	981.28 ± 686.45	877.0 [271–10,002]	[765.5–1101.5]
OGTT—0 MIN (mg/dL)	209	82.69 ± 12.19	80.00 [55–139]	[75.00–88.00]
OGTT—60 MIN (mg/dL)	208	125.19 ± 29.15	121.00 [61–265]	[106.00–145.75]

Abbreviations: BMI, body mass index; OGTT, oral glucose tolerance test; EFW, estimated fetal weight; LMP, start date of the last menstrual period; US, ultrasound; BPD, biparietal diameter; AC, abdominal circumference; FL, femur length.

**Table 3:** Demographic and Clinical Characteristics of Study Participants.

Clinical Characteristic	n (%)
Systemic disease	
Absent	186 (89.0)
Present	23 (11.0)
Smoking	
Absent	181 (86.6)
Present	28 (13.4)
Regular drug use	
Absent	194 (92.8)
Present	15 (7.2)
Family history of DM	
Absent	146 (69.9)
Present	63 (30.1)
Previous pregnancy DM	
Absent	203 (97.6)
Present	5 (2.4)
Preeclampsia	
Absent	201 (96.6)
Present	7 (3.4)
IUGR	
Absent	197 (94.7)
Present	11 (5.3)
Preterm delivery	
Absent	198 (95.2)
Present	10 (4.8)

Abbreviations: DM: Diabetes Mellitus, IUGR: Intrauterine Growth Restriction.

**Table 4:** Comparison of Uterine and Umbilical Artery Doppler Indices Between Two Time Points.

Variables	n	Mean ± SD	*p*-Values
Right Uterine Artery PI 1	209	0.92 ± 0.38	0.189
Right Uterine Artery PI 2	209	0.94 ± 0.40	
Right Uterine PI-Z Score 1	208	−0.17 ± 1.37	0.412
Right Uterine PI-Z Score 2	208	−0.10 ± 1.48	
Right Uterine RI 1	209	0.54 ± 0.11	0.437
Right Uterine RI 2	209	0.55 ± 0.12	
Right Uterine S/D 1	209	2.35 ± 0.78	0.107
Right Uterine S/D 2	209	2.42 ± 0.95	
Left Uterine Artery PI 1	209	0.95 ± 0.31	0.387
Left Uterine Artery PI 2	209	0.96 ± 0.35	
Left Uterine PI-Z Score 1	208	0.08 ± 1.18	0.464
Left Uterine PI-Z Score 2	208	0.11 ± 1.23	
Left Uterine RI 1	209	0.56 ± 0.09	0.566
Left Uterine RI 2	209	0.56 ± 0.10	
Left Uterine S/D 1	209	2.40 ± 0.67	0.375
Left Uterine S/D 2	209	2.42 ± 0.75	
Mean Uterine Artery PI 1	209	0.94 ± 0.28	0.200
Mean Uterine Artery PI 2	209	0.95 ± 0.31	
Mean Uterine PI-Z Score 1	209	−0.05 ± 1.04	0.284
Mean Uterine PI-Z Score 2	209	0.00 ± 1.14	
Mean Uterine RI 1	209	0.55 ± 0.08	0.411
Mean Uterine RI 2	209	0.55 ± 0.09	
Mean Uterine S/D 1	209	2.37 ± 0.58	0.129
Mean Uterine S/D 2	209	2.42 ± 0.68	
Umbilical Artery PI 1	209	1.10 ± 0.17	0.901
Umbilical Artery PI 2	209	1.11 ± 0.18	
Umbilical Artery PI-Z Score 1	208	−0.12 ± 1.06	0.947
Umbilical Artery PI-Z Score 2	208	−0.12 ± 1.07	
Umbilical Artery RI 1	209	0.68 ± 0.06	0.900
Umbilical Artery RI 2	209	0.68 ± 0.06	
Umbilical Artery S/D 1	209	3.26 ± 0.69	0.898
Umbilical Artery S/D 2	209	3.27 ± 0.70	
MCA PI 1	209	1.86 ± 0.37	<0.001
MCA PI 2	209	1.74 ± 0.33	
MCA PI-Z Score 1	208	0.29 ± 1.25	<0.001
MCA PI-Z Score 2	208	−0.18 ± 1.30	
MCA RI 1	209	0.82 ± 0.07	<0.001
MCA RI 2	209	0.81 ± 0.07	
MCA S/D 1	209	6.77 ± 5.30	<0.001
MCA S/D 2	209	5.62 ± 2.46	
CPR 1	208	1.73 ± 0.44	0.004
CPR 2	208	1.61 ± 0.41	

**Table 5:** Comparison of participants with positive and negative 50 g challenge test results in terms of changes observed between the two Doppler measurements.

	50 gr Challenge Test	n	Mean	SD	25th	Percentiles	75th	*p* *
Right uterine PI	Positive	62	−0.0448	0.32783	−0.2325	−0.0400	0.1050	0.477
	Negative	147	−0.0031	0.29899	−0.1700	−0.0100	0.1400	
Right uterine RI	Positive	62	−0.0087	0.09943	−0.0625	−0.0200	0.0525	0.481
	Negative	147	−0.0020	0.09712	−0.0600	0	0.0500	
Right uterine S/D	Positive	62	−0.2031	0.93840	−0.4225	−0.0700	0.2025	0.320
	Negative	147	−0.0154	0.58621	−0.2900	−0.0100	0.2000	
Left uterine PI	Positive	62	0.0673	0.27043	−0.1000	0	0.2125	0.014
	Negative	147	−0.0510	0.27818	−0.1700	−0.0400	0.1000	
Left uterine RI	Positive	62	0.0240	0.08921	−0.0300	0.0200	0.0825	0.013
	Negative	147	−0.0140	0.07742	−0.0600	−0.0100	0.0400	
Left uterine S/D	Positive	62	0.1881	0.72193	−0.1625	0.1050	0.3925	0.004
	Negative	147	−0.1201	0.62294	−0.3500	−0.0600	0.2000	
Umbilical artery PI	Positive	62	0.0155	0.18471	−0.1250	0.0100	0.1625	0.367
	Negative	147	−0.0114	0.19055	−0.1200	−0.0200	0.1200	
Umbilical artery RI	Positive	62	0.0081	0.07310	0	0	0.1000	0.377
	Negative	147	−0.0014	0.07401	0	0	0	
Umbilical artery S/D	Positive	62	0.1653	0.80181	−0.3750	0.0450	0.6650	0.333
	Negative	147	−0.0024	0.74007	−0.3700	0	0.4200	

*: Mann–Whitney U test.

**Table 6:** Comparison of participants with positive and negative 100 g OGTT results in terms of changes observed between the two Doppler measurements.

	Test Result	n	Mean	SD	25th	Percentiles Median	75th	*p* *
Right uterine PI	Positive	7	0.0100	0.18276	−0.1400	0.0300	0.1800	0.662
	Negative	174	−0.0186	0.29681	−0.1900	−0.0250	0.1325	
Right uterine RI	Positive	7	0.0029	0.05407	−0.0300	−0.0200	0.0600	0.897
	Negative	174	−0.0054	0.09539	−0.0600	0	0.0500	
Right uterine S/D	Positive	7	−0.0900	0.38850	−0.3500	−0.1800	0.2200	0.791
	Negative	174	−0.0607	0.65831	−0.3100	−0.0250	0.2000	
Left uterine PI	Positive	7	0.1329	0.15934	−0.0900	0.1800	0.2500	0.042
	Negative	174	−0.0312	0.28155	−0.1700	−0.0200	0.1125	
Left uterine RI	Positive	7	0.0471	0.06576	−0.0400	0.0700	0.0900	0.048
	Negative	174	−0.0086	0.07981	−0.0600	−0.0100	0.0425	
Left uterine S/D	Positive	7	0.2729	0.37518	−0.1900	0.3900	0.4900	0.037
	Negative	174	−0.0703	0.65948	−0.3225	−0.0500	0.2225	
Umbilical artery PI	Positive	7	0.0829	0.13413	−0.0500	0.0900	0.2400	0.176
	Negative	174	−0.0079	0.19181	−0.1200	−0.0100	0.1200	
Umbilical artery RI	Positive	7	0.0429	0.05345	0	0	0.1000	0.135
	Negative	174	0.0017	0.07486	0	0	0.1000	
Umbilical artery S/D	Positive	7	0.2243	0.47017	−0.1500	0.2200	0.7500	0.429
	Negative	174	0.0200	0.74920	−0.3500	0.0100	0.4425	

*: Mann–Whitney U test.

## Data Availability

The original contributions presented in this study are included in the article. Further inquiries can be directed to the corresponding author.
